# Creutzfeld‐Jakob disease: experience in a neurological center from Argentina

**DOI:** 10.1002/alz70858_100211

**Published:** 2025-12-25

**Authors:** Maria Eugenia Sarandria

**Affiliations:** ^1^ FLENI, Buenos Aires, Ciudad Autonoma de Buenos Aires, Argentina

## Abstract

**Background:**

Creutzfeld‐Jakob disease (CJD) is an extremely rare neurodegenerative disorder, caused by the deposition of pathogenic protein components, known as prions, in the central nervous system. It may present a variable combination of symptoms that include rapidly progressive dementia, ataxia, extrapyramidal symptoms, and myoclonus. Its sporadic form is the most frequent (90%). To date, there is no effective treatment, and the average survival time is 5 months. Given its low frequency, there is little evidence on prognostic and therapeutic factors.

**Method:**

Retrospective review of medical records of patients with a diagnosis of CJD based on the WHO diagnostic criteria, who consulted in a referral neurological center in Argentina between January 2006 and July 2021. Data about demographic variables, presenting symptoms, magnetic resonance imaging of the brain, electroencephalogram, protein 14.3.3, treatment and survivance was recorded for each case.

**Result:**

64 patients were included (73% men, median age 64 years). 1 patient had a diagnosis of genetic CJD, the remaining 63 cases corresponding to sporadic CJD. 91% had a probable CJD diagnosis according to the WHO criteria. The most frequent clinical variants were classical (28%) and cognitive (28%). 23 patients received glucocorticoid treatment (35%). The median survival time was 7 months. Clinical presentation and treatment with corticosteroids did not have a significant impact on survival times, while the use of antipsychotics was associated with a notable reduction of it.

**Conclusion:**

In our experience, the median survival time of patients with CJD was 7 months. The use of antipsychotics could lead to a reduction in survival times.

